# Exploring Older Adults’ Adoption and Use of a Tablet Computer During COVID-19: Longitudinal Qualitative Study

**DOI:** 10.2196/32957

**Published:** 2022-03-08

**Authors:** Sunyoung Kim, Willow Yao, Xiaotong Du

**Affiliations:** 1 School of Communication and Information Rutgers University New Brunswick, NJ United States

**Keywords:** older adults, tablet computer, technology acceptance, mental model, longitudinal study, COVID-19

## Abstract

**Background:**

As mobile computing technology evolves, such as smartphones and tablet computers, it increasingly offers features that may be particularly beneficial to older adults. However, the digital divide exists, and many older adults have been shown to have difficulty using these devices. The COVID-19 pandemic has magnified how much older adults need but are excluded from having access to technologies to meet essential daily needs and overcome physical distancing restrictions.

**Objective:**

This study sought to understand how older adults who had never used a tablet computer learn to use it, what they want to use it for, and what barriers they experience as they continue to use it during social isolation caused by the COVID-19 pandemic.

**Methods:**

We conducted a series of semistructured interviews with eight people aged 65 years and older for 16 weeks, investigating older novice users’ adoption and use of a tablet computer during the nationwide lockdown due to COVID-19.

**Results:**

Participants were gradually yet successfully accustomed to using a tablet computer to serve various daily needs, including entertainment, social connectedness, and information-seeking. However, this success was not achieved through developing sufficient digital skills but rather by applying the methods they were already familiar with in its operation, such as taking and referring to instruction notes.

**Conclusions:**

Our findings imply that older adults without digital literacy can still benefit from a digital device for quality of later life if proper traditional methods they are already familiar with are offered in its use.

## Introduction

### Overview

Thanks to rapid technological advancements and decreasing costs, mobile devices are becoming accessible to more older adults, which led the aging population to become the fastest-growing group of technology adopters [1]. The statistics show that internet usage among US residents aged 65 years and older has surged from 43% in 2010 to 75% in 2020 [2], and more than 61% of this population owned a mobile phone as of 2020 compared to only 18% in 2010 [3]. Along with mobile phones, the number of tablet users is also on the rise, with roughly 50% of US adults owning a tablet computer in 2021 [3]. The increased ownership of a tablet computer is particularly promising for older adults since a larger screen size is positively associated with older adults’ technology adoption rate [4]. For older adults with impaired vision and reduced dexterity, a larger screen size and better screen resolution are crucial for device usability. Thus, tablets with a larger screen than mobile phones have been more appealing to senior users [5].

However, the digital divide by age still exists, as only 39% and 18% of older adults own a mobile phone and a tablet computer, respectively [3]. This trend is not different in European countries: 35% of people aged 65 years and older did not own a mobile phone, in contrast to over 98% of those aged 18 to 45 years that did own a mobile phone in the United Kingdom in 2020 [6]. Furthermore, digital literacy to use mobile devices comfortably is negatively related to age [7], and older adults often encounter numerous challenges in using new technologies [8-11]. Socioeconomic status is another crucial factor contributing to the digital divide. Approximately 40% of adults with lower incomes (households earning less than US $30,000 a year) do not have home broadband services or a computer, and a majority of them are not tablet owners [12]. By comparison, each of these technologies is nearly ubiquitous, if not multiple of each of these technologies, among adults in households earning US $100,000 or more a year [12]. When age and socioeconomic status are combined, it becomes evident that low-income older adults are most likely to face a digital divide [13].

The COVID-19 pandemic has magnified the importance but lack of access to mobile devices by older adults [14,15]. The mandated shelter-in-place and social distancing orders transformed most tasks that have been performed through face-to-face means into virtual formats. For instance, local organizations and community associations shifted their information distribution and outreach efforts from offline to online platforms, and in-person meetings and events are substituted with virtual methods of communication. While a shift to digital enabled people to stay connected and informed amid the nationwide lockdown, many older adults who were already experiencing social isolation and loneliness were excluded from digital services, which significantly degraded their quality of everyday lives [16-18]. Consequently, efforts to increase older adults’ access to technology have been spurred by both nonprofits and public agencies.

One such effort was carried out by the Housing Authority of the City of Elizabeth, a low-income housing organization in the greater New York area, to distribute over 100 tablets to low-income senior residents to enhance their access to service and information online in the fall of 2020. Although this effort was well-received by the residents, it raised a subsequent concern about the sustainable use of this technology because most recipients were first-time tablet users. They needed to gain sufficient digital skills and develop technological self-efficacy to use a tablet.

We conducted weekly semistructured interviews for 16 weeks with eight senior residents who received a tablet computer to serve two goals: (1) practically, to help the recipients learn digital skills to comfortably use a tablet; and (2) theoretically, to investigate how older novice users learn and develop digital skills over time. From this study, we aimed to answer the following research questions:

1. What do older adults use a tablet for during COVID-19?
2. What challenges do they experience when using a tablet, and how do they progressively cope with those challenges?
3. What are the factors that affect older adults’ learning of digital skills?

To date, the prospect of older adults’ learning to use mobile devices has been extensively discussed in the literature. However, most studies relied on self-reports of past experiences [19,20] or quasiexperiments on various learning modes [21,22]. This paper contributes to the literature by investigating, for 4 months, the real-world context of how older adults who are new to tablet technology progressively learn digital skills to perform desired tasks on a tablet.

Our findings revealed that our participants were willing to learn and successfully use a tablet for entertainment, social connectedness, and information-seeking purposes as the study proceeded. However, it was not through acquiring sufficient digital skills, despite their continuous learning endeavor, but through incorporating the method they are already familiar with in its operation: pen and paper. The underlying issue with difficulty in acquiring digital skills was the lack of a proper mental model of how a tablet works. These findings can be used as design guidelines to promote the sustained use of emerging personal technologies to support the aging society. To the best of our knowledge, this is the first study that investigated the progressive use of a tablet computer among novice older users through a longitudinal field deployment study.

### Literature Review

#### Background

Digital literacy is a set of skills and knowledge required by individuals to use digital devices to access and use digital information effectively [23]. As today’s young adults who are savvy users of current digital devices become older, they will not experience much difficulty in using them. However, the digital divide will continue to exist because computing technology continues to advance, and new digital devices will continue to emerge. Perhaps one of the earliest research topics on older adults’ technology use was investigating their use of automatic teller machines in the late 1990s [24]. Moreover, researchers are still investigating older adults’ use of emerging technologies (eg, wearables [25], voice assistants [26]).

Information and communication technologies are becoming an integral part of our everyday lives as information and services are increasingly delivered and consumed online [27]. In particular, access and use of mobile devices such as smartphones and tablets are vital rather than an option, since these are used as a primary gateway to obtain needed information and services for many daily tasks [28]. Consequently, a growing number of older adults are expected to use mobile devices to fulfill some of their everyday needs. However, unlike younger generations, older adults did not grow up with technologies that are prevalent today. Many older adults are not familiar with new technologies and thus have difficulties in using them. Therefore, there has been an emerging focus on helping the aging population learn to use mobile devices.

#### Older Adults and Technology Acceptance

Much research has sought to comprehend technology use and acceptance by older people using existing theories of technology adoption such as the technology acceptance model (TAM) [29] and the unified theory of acceptance and use of technology (UTAUT) [30] (eg, [31-33]). Although these theories are widely used to evaluate user attitudes toward the acceptance of technology in general, limitations exist because they lack sufficient consideration on age-specific or age-related factors. To overcome such limitations, researchers have extended these theoretical frameworks to specifically account for older adults’ technology acceptance behaviors [8,11,34-36]. Details may differ in these extended frameworks, but one common factor that distinguishes older adults from their younger counterparts in technology acceptance is the special learning needs that older adults have when using new technology.

For example, Renaud and van Biljon [35] postulated the senior technology acceptance model (STAM) expanding on TAM [35]. STAM introduced an incorporation phase in which older adults explore and experiment with new technology to estimate the perceived ease of learning and use for technology acceptance. Similarly, Kim et al [34] developed a theoretical model that extends TAM and UTAUT to explain how older adults accept or reject mobile devices. This model comprises an additional phase, intention to learn, with three affecting factors (self-efficacy, conversion readiness, peer support) that determine the acceptance of mobile devices among older adults. They further validated this model through a comparative study with younger adults [37]. Third, Barnard et al [8] proposed a model of technology acceptance and rejection for older adults, which emphasized the facilitating conditions to learn a technology, such as a friendly space for trial and error and provision of a manual, as a decisive factor for seniors’ technology adoption.

As learning has been found to be crucial for older adults’ technology acceptance, researchers have sought ways for older adults to effectively learn to use new technology, which we explain in the next section.

#### Older Adults’ Learning to Use Mobile Devices

Researchers have investigated various modes of learning to determine older adults’ preferences when learning to use mobile devices. For instance, Pang et al [21] conducted a design prove study of an interactive help kiosk, Chiu et al [38] conducted a focus group of a small-group tutoring approach, LoBuono et al [39] observed the utility of intergenerational learning by college students, and den Haan et al [40] ran a field study of peer-to-peer community learning. Although findings from these studies were mixed as to which learning methods older adults preferred or found effective, they share a common preference: a collaborative approach implemented by peer support, interpersonal communication, and community-based programs. In contrast, independent learning was preferred for its ability to control learning speed and avoid bothering family and friends, although its effectiveness is limited.

In summary, mobile devices are becoming essential for healthy aging and independent living, but many older adults experience various challenges with respect to using new technology. Learning is imperative in overcoming those challenges, although empirical evidence on how older novice users learn and develop digital skills for their sustained use of mobile devices is limited. This paper contributes to this body of literature by exploring what challenges older novice users experience when learning to use a tablet computer and how they cope with those challenges over time through a longitudinal field study.

## Methods

### Ethical Considerations

The study was approved by the Rutgers Institutional Review Board (Pro2020002565), and informed consent was obtained from all participants before participating in the study.

### Participants

For participant recruitment, we collaborated with the Housing Authority of the City of Elizabeth, an affordable housing community located in the greater New York area that the first author has a long-established research collaboration with. This community manages 1322 public housing units of various types, including single units, family housing, and senior complexes, approximately 1000 of which are for a low-income senior and disabled population. To improve older adults’ access to technology during COVID-19, this organization raised a fund to distribute free tablet computers to senior residents. As a result of this effort, about 100 senior residents in this housing community received a free tablet in the fall of 2020. Among those who received a tablet, we recruited participants to take part in this study. When giving out a tablet, a recipient was asked if they were interested in participating in this study. Upon agreement to participate, we installed the Zoom app on their tablet before giving it out. Three inclusion criteria for participation were age being over 65 years, English-speaking, and being a first-time tablet owner.

In total, we recruited 10 participants (6 females and 4 males), ranging in age from 65 to 80 years (mean age 71.6, SD 4.9 years; see Table 1). Nine participants lived by themselves and one participant lived with a partner. With respect to general technology use, six participants owned a smartphone, four participants owned a flip phone, six participants owned a computer, and one participant owned an eBook (Kindle). All participants said that they frequently used computers for information searching and email. Seven participants said they had used a tablet but never owned it, and three said they had never used a tablet. The self-identified ethnicity of all participants was Black or African American. Two participants withdrew within the first month of the study due to losing interest in the study. The other eight participants completed the study for the entire study duration.

**Table 1:** Participant demographics.

Participant ID	Age (years)	Gender	Devices owned	Occupation before retirement	Study duration
P1	65	Male	Smartphone, computer	Machine operator	Completed
P2	66	Female	Flip phone, computer	Nurse aid	Completed
P3	71	Female	Smartphone	Customer service	Completed
P4	80	Male	Smartphone, computer	Tax preparer	Completed
P5	74	Female	Smartphone, computer	Paraprofessional	Completed
P6	68	Female	Flip phone	Customer service	Completed
P7	72	Male	Smartphone, computer	Sales	Completed
P8	78	Female	Flip phone, computer, eBook	Homemaker	Completed
P9	69	Male	Smartphone	Sales	Dropped
P10	73	Female	Flip phone	Customer service	Dropped

### Data Collection

Participants were told to freely use a tablet as much or as little as they wanted throughout the study period, and we conducted a weekly semistructured interview for 16 weeks between fall 2020 and spring 2021. All interviews were conducted virtually via the Zoom video-conferencing app. For those who did not know how to use Zoom, we conducted the first interview over the phone and provided step-by-step instructions for launching the app and then switched to Zoom for the rest of the first interview.

Our interview protocol consists of two sessions: the first half of each interview explored participants’ daily use of a tablet and their reflection on its use, and the second half was devoted to providing in-person instruction on using a tablet for various features. For the first session, we investigated what older adults wanted to use a tablet for, what difficulties they faced when using it, and how they coped with those difficulties. To explore these spaces in different stages of use, we constructed three sets of open-ended interview questions by phase. In the first phase (weeks 1 to 4), we focused on understanding initial impressions, needs, and difficulties in using a tablet. In the second phase (weeks 5 to 14), we explored the user experience in-depth, including usage patterns, the needs and challenges, and strategies to cope with breakdowns. In the third phase (weeks 15 and 16), we focused on the overall reflection on users’ interaction with a tablet. Each session lasted from 30 to 60 minutes. Next, for the second session, we asked participants if they wanted to learn any feature and provided step-by-step instructions for the feature. We did not prepare any instruction material but provided impromptu verbal explanations on how to perform a requested task. This session lasted about 30 minutes.

In addition, participants filled out a short survey to inform us about their basic demographic information, including age, household type, occupation before retirement, and devices owned. All interviews were audio-recorded and transcribed. Participants who completed the study were compensated with a US $160 gift card upon completion. Those who withdrew were partially compensated, and the amount was prorated by the duration of participation.

### Data Analysis

We analyzed our interview data using thematic analysis to reveal patterns across data sets. Thematic analysis is a method for identifying, analyzing, and reporting patterns and themes within qualitative data [41]. We selected thematic analysis because it enables investigating explanatory conceptual themes associated with older adults’ use of a tablet over time. The thematic analysis process involves open coding, axial coding, and selective coding for theme identification.

First, we conducted open coding to identify concepts that are significant, such as abstract representations of events, objects, happenings, and actions that emerged from the data. For open coding, each author separately created a set of codes for a selected interview transcript. All authors then met and compared a list of individually generated codes to validate, cross-validate, and consolidate the codes. We iterated this process four times when we were convinced that the coding process had saturated. Using the consolidated codebook, the first author then coded the rest of the interview transcripts. Next, we categorized the related concepts created by open coding into conceptual phenomena using axial coding. Phenomena refer to repeated patterns of events, happenings, actions, and interactions, representing people’s responses to problems and situations. Lastly, we followed the selective coding process to integrate all concepts extracted from axial coding into a single storyline through building relationships.

## Results

### Overview

In the first interview, all participants said that they used to engage actively in the events and activities organized by a local senior center before COVID-19, which constituted most of their physical activities and social interactions. Thus, unsurprisingly, they all expressed increased feelings of loneliness and social isolation due to social distancing and shelter-in-place orders during COVID-19.

I used to go to the senior center every day and play games. I can’t do that anymore. My daily routine now, because of the pandemic, is not a real good routine because I am home all day. The biggest joy I have in life now is going to Shoprite and buying my groceries once a week. And we wave at each other if there are any of my friends there.

Participant (P) 2, Interview (W) 1

Before the COVID-19 hit, I was part of the senior center where I used to go four times a week. I was into the ceramics classes, exercises, and all kinds of different activities at the center. Now, I’m always in the house.

P6W1

I’m so used to going to the senior center. Now we can’t go to the center. Now I have a tablet, so I could still play bingo and talk to my friends. But being in person is much better for us so that you can get up and talk to people and have refreshments.

P7W1

Fortunately, most participants quickly acknowledged that a tablet could serve as an outlet for sustaining a quality life and remaining connected with other people amid COVID-19 within the first couple of weeks of the study. Consequently, they tried to make good use of a tablet to fulfill various needs throughout the study. While many of these efforts were successful, several participants failed to gain sufficient digital literacy to comfortably use a tablet even upon completion of the 4-month-long training. In what follows, we report the findings on our participants’ purposes, perceived benefits, and challenges of using a tablet. While these are not much different from tablet use in general, our findings show empirical evidence of older novice users’ adoption and use of a tablet over time.

### Purposes and Benefits of Using a Tablet

#### Entertainment: Playing Mobile Games

The most common topic of questions about tablet use in the first phase of the study was how to install mobile game apps. Some participants were already playing mobile games on their smartphones and asked to help install the same apps to their newly received tablets, and others asked how to find and install new mobile games on a tablet. Similarly, the most common use of a tablet was for playing mobile games. To our opening question of each interview, “what did you use your tablet for this past week?”, the prevalent answer throughout the study was playing a mobile game.

Before COVID, I didn’t play the game, because I didn’t have a tablet. Since I have my tablet now, I’m playing lots of games. Because of COVID, I am forced to sit down and learn things that I didn’t have to do before, like playing a game. Now, even if we start going back out, I will probably carry my tablet wherever I go and use it.

P2W8

People usually play mobile games to spend leisure time or alleviate boredom [42]. However, our participants did not mention any of these as their intention of playing mobile games. Instead, they emphasized and made clear that they played mobile games for constructive purposes such as engaging in challenges, gaining a feeling of accomplishment, and keeping active mentally and physically. We are uncertain if the noted intentions were genuine or due to a response bias where a respondent exhibits the purposeful presentation of self to fit into socially desirable attitudes or please an audience [43]. What we are certain of is that our participants were conscious of the potential benefits that playing mobile games can bring to them. The downside is that they played only a couple of simple games, even though numerous mobile games have been designed for cognitive, physical, and hedonic benefits for healthy aging. Although the excerpts below mentioned some other games, Bingo and Scribble were two games that all participants stated they played throughout the study.

I play a few games every day like Bingo, Scribble, and Candy Crush on my tablet to keep my brain active and my coordination back and forth. I try to keep on challenging myself with the games.

P3W2

I like to play a challenging game on a tablet, like three-word matching games, because I had many strokes. Playing games keeps my brain active, keeps your eyes and hands move, and it makes you keep thinking.

P7W3

I have an app called Happy color on my tablet that I play all the time. It has all different pictures on it, and you just color them by the number that’s noted. I love to play that because it’s comforting and soothing to me.

P8W4

In general, the entertainment purposes of using a mobile device are fulfilled by various activities, ranging from playing games to listening to music, watching TV and videos, and reading books [42]. However, none of these, other than playing mobile games, was brought up by our participants throughout the study, except for one participant mentioning their attempt, but failure, to install a TV app in week 12. In fact, we introduced and provided instructions for using many recreational features mentioned above during the interviews, and their initial responses to these features were positive. However, we had not received any comment about using them throughout the study.

I tried to install a TV app, Pluto TV, on my tablet. But I wasn’t successful because they asked for a password, and I didn’t know what password I have.

P5W12

#### Social Connectedness: Video Chats and More

Because all interviews were to be conducted virtually via Zoom, a video-chatting app, we offered instructions on using it during the first interview until participants fully understood how to use it. They then used Zoom at least once a week to participate in this study for 4 months. These efforts must have enabled our participants to utilize Zoom comfortably and effectively as the study proceeded. Participants unanimously expressed excitement, satisfaction, and a positive outlook for using Zoom to socialize with others and overcome loneliness during COVID-19 throughout the study. Although video-chatting platforms cannot substitute for face-to-face interaction, they still provided those who felt a loss of connection with a way to connect with friends and loved ones safely.

I was by myself for 14 days. The only time I can talk to anybody now is when I zoom on a tablet earlier today. It was a nice experience because we saw more people. It was keeping me from being lonely. It’s helping me stay in touch with people and to get things done. Now it’s another part of my life that I’d like to keep using.

P6W3

We haven’t been in person since we closed down in March. So that was very instrumental in getting these tablets. The most important thing I’m having a tablet for is being able to reach out and talk to other people. This tablet is like my best friend now. He goes with me everywhere. I got to give it some name.

P7W4

I was all by myself. But once I was on the Zoom, it has affected my life tremendously. I can see the seniors who I used to see at the center. A lot of seniors are homebound and very lonely. So, just to be able to be in touch with the other seniors is very meaningful to us. Seeing them is a way of alleviating a lot of the loneliness from being alone.

P3W5

Another common purpose of using Zoom was religious socialization, which is an essential part of many participants’ social activities. One of the popular answers to our interview question “what did you use your tablet for this past week?” was attending virtual church services.

I zoom for my church services on Sunday. I haven’t gone back to my church yet because the pastor had a COVID and came back, but they weren’t taking temperatures or social distancing. But every Sunday, you can watch on zoom the pastor live-streams. You can’t see anybody but the pastor, but you still receive whatever he’s preaching about.

P1W4

A key concern or complaint that participants had with using Zoom was that many other seniors were still unfamiliar with or did not know how to use Zoom. Our participants gained sufficient in-person training and step-by-step guidance on using Zoom from participating in this study. However, many older adults lack access to these resources. Since having more people on video-chatting platforms is essential for virtual socialization, participants sought ways to help other seniors use Zoom.

There’s a lot of seniors that have a tablet but just don’t zoom. I wanted to host a zoom meeting with some of the seniors I know for some of those classes. How do I do that? Do they charge you for that?

P1W5

Today was a good meeting (for the senior center’s class). We had almost 16 seniors, which was phenomenal. As time goes by, we’re getting more on the zoom meetings. We still have some that are having a problem getting on and still don’t understand. So, someone has to go to their house and show them how to get on.

P6W7

The lady who just moved in upstairs was by herself. Last week, I came down to help her get on our Zoom meeting. The lady was really depressed when we spoke to her. She broke down and was crying on zoom. We all told her that we’re all here for her and will call her if necessary. That made her feel much better, and I felt much better too.

P7W10

There are many video-chatting platforms other than Zoom, namely FaceTime, Skype, Google Meet, Microsoft Team, WebEx, and many more. Banskota et al [44] showed that older adults used FaceTime and Skype most frequently while in isolation during COVID-19. However, our participants only used Zoom throughout the study and nothing else. We consider three factors that must have contributed to their heavy reliance on Zoom for virtual socialization: (1) they learned how to use Zoom but no other apps and (2) FaceTime and Skype are generally used among friends and loved ones [44], whereas (3) our participants mostly connected with other seniors via virtual meetings organized by a senior center or a church for which Zoom is the primary tool used.

Besides, our participants fulfilled their needs for social connectedness not only by directly talking to others via video-chatting apps. Equally valuable was to share meaningful and helpful information with friends and loved ones. We received numerous questions on how to send digital data of various formats (eg, pictures, messages, news) to other people throughout the study. Even though it was an indirect experience, sharing digital data still gave older adults a sense of being connected to and engaging with others.

I want to send a picture I take on the tablet to other people. How do I do that? Can you teach me how to send them to like my granddaughter?

P4W4

I love sending inspirational cards to my friends in the morning. I send them wonderful things to uplift everyone’s spirit. Doing that makes me feel connected to them.

P2W5

Someone emailed me the vaccine information. And I wanted to share it with my friend, and so I forwarded it to her and said, you just click on the link. But she couldn’t find the link in my email. I didn’t know how to give her the live link.

P3W8

#### Information Seeking: Google and YouTube

Participants did not ask much about what to use a tablet for when they were first introduced to it, except for playing mobile games and using Zoom. Thus, we spent a great deal of the second session of the first-phase interviews introducing various apps, including but not limited to Google and YouTube, and explaining how to use them.

Participants started mentioning their information-seeking experiences after a few weeks of the study had lapsed. As the study proceeded, participants spent more time sharing their experiences of searching and retrieving various types of information when answering our interview question “what did you use your tablet for this past week?” The topics of information participants searched for encompassed an extensive range of informational needs and everyday activities, ranging from cooking to gardening, health care, home improvement, food, shopping, and many more.

I like looking at different types of cooking on YouTube. This morning, I went to YouTube and put “how to cook turkeys” in the search bar. And it brought up a lot of different ones. What I liked is that I can set my tablet up on the counter and follow the recipe from the tablet. I’ve never done that before. I used to make a copy and follow it.

P8W6

I use my tablet every day nowadays to check out different sites. For example, Facebook gives me information about food distribution, vaccine, testing, stimulus checks, etc. Last week, I looked up Google to see if I could take the COVID shot while rehabbing from having a flu shot. So that was a good resource to find out.

P7W9

Whenever I need information about my gardening, I take the tablet and put a question into Google, and I get my answer. The other day when a branch broke off from one of my blueberry bushes, I went into Google to find out how to replant it.

P1W10

We identified two interesting aspects of our participants’ tablet use for information-seeking. First, in contrast to many entertainment features that we introduced but were rarely used, participants quickly adapted to and increasingly used the features for information-seeking as the study proceeded. We attribute this to older adults’ substantial informational needs for independently managing everyday life as much as their impending emotional needs for socialization. Second, although the general purposes of using YouTube range widely from information-seeking to education, entertainment, exercise, and more [45], participants used YouTube only to retrieve practical information. We introduced YouTube as “a place where you can watch videos online” to participants; nevertheless, they watched YouTube videos only to fulfill their informational needs. Many possible reasons can account for this usage pattern (eg, lack of interest, unfamiliarity with browsing, not knowing what is available), although our data did not present any concrete reason. Further studies are required to examine older adults’ use of YouTube.

### Challenges and Coping Strategies for Tablet Use

#### Dealing With Challenges Due to Forgetfulness

The most prevalent difficulty that all participants experienced and asked for help with was managing passwords. In the first few interviews, participants spent most of the second sessions asking for help logging in to different apps and websites. Some participants could not log in to certain services because they did not have an account, and some could not because they did not remember their password, all of which we quickly resolved by helping them create a new account or find a password. What we struggled with the most was when the password a participant remembered did not work. We asked them to retry multiple times, which made them feel confusion, frustration, and decreased confidence. Some services became deactivated after several login trials, and thus we helped them proceed with extra steps for reactivation. However, many of them gave up after a few failed attempts and told us that they did not want to use it anymore. After several similar incidents, we realized that the password participants remembered was, in fact, for a different service in most cases (eg, using a tablet’s passcode for Google login).

I have a password that I set up, but it keeps telling me it’s the wrong password. So, I have to check into that. I know that’s what I set up but don’t know why it’s not accepting it. I probably had to do forgot password and set up another one.

P4W2

As the study proceeded, participants gradually got used to managing multiple accounts and passwords through repeated instructions, although not through memorization. Except for relying on the autocomplete feature, a common strategy was to keep written records of accounts and passwords. In fact, writing down passwords is an old and widespread practice [46,47], and some of our participants already kept a written record of their passwords. We observed that novice participants also quickly developed a practice of keeping their newly created account information on paper and resorted to written copies of passwords for logins. However, its effectiveness and sustainability were questionable.

People tell you don’t use the same password. So, I have so many different passwords. And I have a diary with all of my passwords in it. I have to see the diary to find the password so that I can do whatever I want to do.

P2W2

When I create an account, I write its password on a piece of paper. And then I lay the paper over here or over there, and I don’t use it for long. And then when I have to use it, I don’t know what the password was. And by then, the paper is gone.

P3W2

One effective way to extend this practice is to digitize written records, as mentioned by P1 in week 4, although nobody else mentioned this.

I wrote all my passwords and the names of apps on a piece of paper and took a picture of it. So, if I need to find a password, I go to my photos gallery and pull up my password and the name of the app.

P1W4

Unsurprisingly, none of our participants was aware of any password-managing tool, and we did not consider introducing it to them either, because it was too complicated for them to use. In addition, privacy and security concerns relating to keeping written copies of passwords were never brought up, which confirms prior work showing that older adults are unaware of and susceptible to privacy and security risks associated with technology use [48].

We also observed that keeping written records of information was used not only for managing passwords. Most participants kept written instructions for various features we taught and counted on those whenever needed (eg, adjusting screen brightness, changing font size). In all, participants relied heavily on a traditional pen-and-paper method to cope with their forgetfulness throughout the study.

The only thing that bothers me is that I can’t get the tablet connected to my printer. I’m so used to making copies of instructions. Do I need some type of a cord to connect a tablet to the computer and then print?

P5W3

I wrote down the steps of changing the font size on stickies. Stickies are now all over my place. Until I get more familiar with how to do it, I need to follow the notes.

P3W7

When I find a recipe on YouTube, I sometimes want to print it out. But it didn’t show me where I can make a copy. I didn’t want to lose it and having to start all over again. But I don’t know how to print it out from my tablet.

P6W6

#### Dealing With Challenges Due to Unfamiliarity With New Technology

Because most participants had never used a tablet before, we anticipated that a lack of self-efficacy, one of the most significant psychological challenges for older adults’ technology adoption [34], would prevail, at least in the first few weeks of tablet use. As expected, participants reported many experiences of having a fear of making mistakes or not reversing them after making mistakes.

The thing I’m afraid of the most with the tablet is that I may hit the wrong button because I didn’t want to mess up something. The other day, I was writing an email. And maybe I hit something that I was not supposed to, and lost the email. I didn’t know how to get it back. Someone said draft or something, but I don’t know where to find it.

P3W3

When she (a friend) sent me an email, I could barely read it because it was so small. So, she explained to me how to enlarge it. Then, it became so large that it overruns the page. But I didn’t want to go back and change the font because I didn’t have confidence that I was proficient enough to change font size without making a mistake. What if I hit the wrong something and mess up everything?

P7W4

The second prevalent topic that participants asked for help with, following managing passwords, was adjusting the settings on a tablet and apps, such as display brightness, font size, screen timeout, volume, and screen orientation. In contrast to the questions about password management, which gradually decreased in frequency as the study proceeded, the questions about controlling the settings persisted throughout the study. Unfortunately, we did not find any evidence of participants becoming used to managing most settings, even after 4 months of training. As mentioned previously, most participants resorted to step-by-step instruction notes they wrote down on paper when adjusting settings. Furthermore, whenever participants tried a new app, they experienced similar difficulties setting it up and asked for help even in the later phase of the study.

I was trying to play a new mobile game, but its screen was vertical. I wanted horizontal. At one point, it did fine. But then I got stuck in vertical. And I couldn’t get it out of vertical when I played the came next day. Is there a way to change it?

P1W8

Today, I went on Zoom for a chair exercise. There were five people on including the instructor. All of our boxes were pretty big. I wanted to make them smaller so that I could see the instructor bigger. But I didn’t know how to resolve that.

P5W11

Even though participants counted on written notes for most operations, their self-efficacy still improved significantly as the study proceeded. Regardless of relying on written notes, successfully operating intended functions for themselves increased their confidence in using a tablet, which positively influenced their willingness to try new things on a tablet. For this, repeated trials were mandatory, which participants considered not a burden but a process.

I’m in my 70s, and this was the first time that I managed to be on Zoom for myself. I think three or four times will make it better. I’m not grasping things as fast as I used to. I have to do it many times to be more competent in what I’m doing.

P3W2

When my friend was trying to teach me how to put Zoom on my phone, she just gave up. Finally, I am on Zoom now!

P4W2

I felt very nervous (of using a tablet). But as it went along, I did pretty good. I still would like to have more practice so that I feel more confident within myself. After you get a certain age, you don’t retain a lot of things, and a lot of things are harder to do. And when you start something, and you conquer it, it makes you feel good. I haven’t conquered the tablet, but I’m not as afraid to use it as I was initially.

P5W13

In the last interview, most participants expressed gratitude and satisfaction with participating in this study for having an opportunity to gain in-person instructions on using a tablet for an extended period. However, this approach is not scalable or widely available. Our participants proceeded with repeated trials supported by our research team by participating this study, but many older adults lack access to this type of support.

It was a blessing that I had the opportunity to work with. I learned a lot from this 4-month program with you. You are patient which was great for a senior so that they felt comfortable in trying to learn in this stage of their life.

P8W16

## Discussion

### Principal Findings

Extensive research has sought to elucidate the challenges older adults face when adopting new technology. Consequently, various factors were identified and discussed, such as perceived usefulness, usability, affordability, compatibility, accessibility, reliability, support availability, learning efforts, and self-efficacy [49]. Our study provided a unique and lived environment where low-income seniors who had no prior experience with a tablet received one for free and gained support to use it to fulfill essential needs during the nationwide lockdown due to COVID-19. This unique circumstance eliminated many of these challenges and magnified two factors: learning efforts and self-efficacy. Our findings show that participants gained sufficient self-efficacy to use a tablet comfortably, not by acquiring digital skills but rather by developing ways to address the challenges. This section discusses a possible reason for their difficulty in obtaining digital literacy despite continuous learning endeavors and a strategy to manage the challenges.

### Difficulty in Learning: Lack of a Mental Model

Prior work shows that older adults are able and willing to learn how to use new digital devices [19,34,50]. Our findings confirm that our participants were eager to learn to use a tablet, and successfully used it primarily for entertainment, social connectedness, and information-seeking purposes. However, they did not acquire digital skills to retain and transfer despite repetitive instructions. We consider one reason for this issue to be related to our instruction not guiding to construct a proper *mental model* of how a tablet works.

A mental model refers to “some kind of understanding of how a device works in terms of its internal structure and processes” [51]. Prior research demonstrates that having a proper mental model improves performance on learning and retaining the operating procedures for an unfamiliar piece of equipment [52]. A key to constructing a mental model is to make the learning experience meaningful: the more “meaningful” a learning experience is, the faster it is learned and the better it is retained [53]. In contrast, rote learning focuses on delivering fragments of knowledge, often in isolation from any context [54].

The mode of instruction we offered to our participants was close to rote instruction, delivering the actions required to perform a task (eg, a sequence of buttons to press) without descriptive information of how a device works. In fact, this type of instruction is common in describing how to operate a digital device. For instance, below is a standard instruction for adjusting a mobile device’s font size, which we used to help participants change the font size on their tablet screen. This instruction can be informative to those who have a basic knowledge of how a device works.

1. On your tablet, open the Settings app

2. Tap Accessibility > Font size

3. Use the slider to choose your font size

We initially had a naïve approach of using this rote instruction mode to teach our participants to use a tablet. Later, we realized that this instruction did not contain “meaningful” information for our participants to retain. Our participants, novice older users who were even foreign to some of the terms used, had difficulty understanding, following, and memorizing the instruction. They often needed extra explanations, such as the location of an action item or how to operate a stated action.

**Figure 1:**
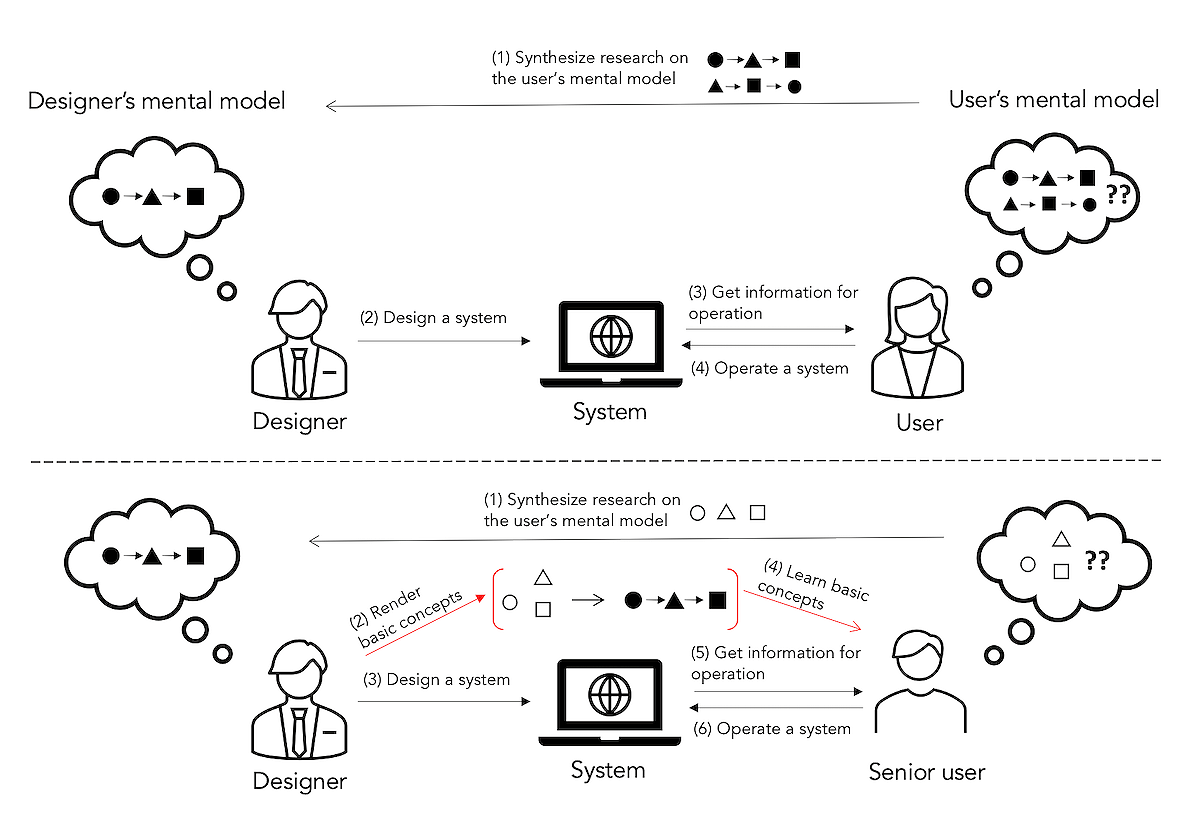
Top: Norman’s conceptual modeling process ([55], page 16). Bottom: a proposed conceptual modeling process for a designer and a senior user. To support a senior user who lacks a basic mental model of a digital device, the designer should first render basic concepts based on a synthesis of research and then design a system. A senior user should first learn basic concepts and then use the system.

The need to bridge the gap in mental models between users and designers is well documented in the fields of human-computer interaction [55]. Research has shown that this gap can be closed by synthesizing a user’s mental model in design. However, this model is not applicable to those who are void of any mental model, such as older novice users. Although various learning modes have been investigated for older adults’ preference and effectiveness [21,39,40], little has been sought to support senior users to construct a basic mental model of how a device works. In consequence, we propose an additional phase in the conceptual modeling process for older novice users: a learning phase through which a senior user can obtain a basic concept of how a system works (see Figure 1). To implement this, not only senior users need to learn the basic concept, but also designers need to render and offer the basic concept that older adults can learn from.

### Solution to Cope With Difficulties: Facilitating Traditional Methods

Efforts have been devoted to developing new digital devices to support the aging population. However, for many older adults, what they are already familiar with might be the best tool to use without fear of making mistakes, a burden to ask others for help, or stress in learning how to use it.

As the study proceeded, all participants increasingly used a tablet comfortably to perform the various activities they intended. However, we observed that this success was not through gaining sufficient digital literacy. While our research team’s in-person instruction must have contributed to their increased tablet use somehow, the most helpful method we observed was to count on what they were already familiar with: pen and paper. Instead of struggling with comprehending and retaining information of using a tablet, participants took notes of necessary details from our instruction and used them later when needed. Despite its scalability and sustainability concerns, pen-and-paper was the easiest, fastest, most efficient, and most reliable method of support for our participants in using a tablet.

Emerging technologies have tremendous potential to support the everyday activities and independence of older adults. However, such potential can be realized only when older adults use them. Because they are exposed to new technology at the later stage of their lives, it is inevitable that they are not familiar with and thus need to learn today's digital devices. While we believe older adults can better understand and retain knowledge to use a digital device once they construct a mental model, effort is still required to learn how to use it. Therefore, we argue that it is important to deliberate on incorporating existing methods that older adults are already familiar with into the design of new digital devices. An excerpt below demonstrates how one participant easily uses GoGoGrandparent, a call-in rideshare support service for transportation.

I have the app (for a ride) that I use all the time but it's not Uber or Lyft. It is called GoGoGrandparent. All I have to do is call them, and they ask me if I want someone to pick me up at my home. If I do, press one and then they tell me how long it will be before an Uber driver gets to my house and what kind of car they're driving.

P7W11

In the end, the goal is not to make older adults learn to use a digital device but to make their lives of better quality. While researchers have sought to enhance older adults’ digital literacy to use a digital device, our findings demonstrate that older adults can benefit from what a digital device offers without much digital literacy if they can integrate a method they are already familiar with in its operation, at least in the short term. Increased use of a digital device will eventually lead to improving digital literacy. Hence, more research is needed to determine how to incorporate the methods older adults are familiar with into designing new technology.

### Limitations and Future Work

Our findings must be evaluated within the context of several limitations. First, we used convenience sampling by recruiting participants from a low-income senior-housing community in an urban region of the United States. Thus, our participant pool may not represent the aging population in general. Selection bias or possible homogeneity of participant characteristics (eg, location, culture, socioeconomic status) might have influenced the responses in the interviews. Second, we acknowledge that our findings might not be exclusive to older adults. However, we did not conduct any comparative study between people in different age groups. Thus, we do not have any evidence to argue whether people in other age groups might encounter a similar learning process as experienced by our participants. Lastly, all participants used the same model of a tablet, whose interface design might have influenced user experience.

We believe our findings could be generalizable to older adults’ adoption and use of any personal computing technologies (eg, computer, smartphone). However, we are cautious of overgeneralization because we did not validate our findings on other technologies and thus lack scientifically significant evidence to argue its generalizability. Hence, the next step is to examine the generalizability of our findings by conducting similar studies on other technologies and through an extensive literature review of relevant studies.

### Conclusion

As technology becomes an integral part of our everyday lives, older adults are increasingly expected to use digital devices to access information and services. Now, the COVID-19 pandemic brought needed attention to a long-standing problem: a digital divide that puts technology out of reach for many seniors, which significantly increased loneliness and social isolation among older adults. As a collaborative effort with a local community organization, we distributed tablets to low-income senior residents to help them access essential services and needed information online during the nationwide lockdown in the United States. This study aimed to serve two goals: practically, to help the recipients learn digital skills to use a tablet, and theoretically, to investigate how older novice users learn and develop digital skills to use a tablet comfortably over time. The findings demonstrate that our participants were willing to learn and successfully use a tablet for entertainment, social connectedness, and information-seeking purposes. However, it was not through acquiring sufficient digital skills but rather by incorporating the method they are already familiar with in its operation. We consider, among other things, that the lack of a proper mental model of how a tablet works prevented them from building digital skills despite repetitive instructions. We are hopeful that our results will encourage future studies to reduce the digital divide and improve the aging population’s access and use of emerging digital devices for a better quality of later life.

## Abbreviations

STAM: senior technology acceptance model
TAM: technology acceptance model
UTAUT: unified theory of acceptance and use of technology
